# Risk of Atherosclerotic Cardiovascular Diseases in various Ethnicities of Pakistan

**DOI:** 10.12669/pjms.36.6.2618

**Published:** 2020

**Authors:** Tariq Ashraf, Asif Nadeem, Shakil Sarwar, Musa Karim

**Affiliations:** 1 Tariq Ashraf, National Institute of Cardiovascular Diseases (NICVD), Karachi, Pakistan; 2 Asif Nadeem, National Institute of Cardiovascular Diseases (NICVD), Karachi, Pakistan, Combined Military Hospital (CMH) Malir, Karachi, Pakistan; 3 Shakil Sarwar, National Institute of Cardiovascular Diseases (NICVD), Karachi, Pakistan, Combined Military Hospital (CMH) Malir, Karachi, Pakistan; 4 Musa Karim, National Institute of Cardiovascular Diseases (NICVD), Karachi, Pakistan

**Keywords:** Atherosclerotic Cardiovascular Diseases, Risk Assessment, Pooled Cohort Equations, Ethnicities, Pakistan, Astro-CHARM, Primary Prevention

## Abstract

**Objective:**

To estimate the risk of atherosclerotic cardiovascular diseases (ASCVD) in various ethnicities of Pakistan using Astronaut Cardiovascular Health and Risk Modification (Astro-CHARM) risk calculator and the Pooled Cohort Equations (PCE).

**Methods:**

Individuals of both gender between 40 to 65 years of age without pre-existing coronary artery disease from residents of Karachi were recruited through snowball sampling technique. Study was conducted at the National Institute of Cardiovascular Diseases, Karachi, Pakistan during January to June 2019. Ethnicity of the participants was categorized based on their mother tongue. Ten-year risk of ASCVD event was estimated using Astro-CHARM Calculator and PCE.

**Results:**

Mean age of a total of 386 individuals was 49(±7.1) years and 45.3% (175) were females. Urdu-speaking individuals were 33.4%(129) of the sample, while, 18.4%(71) Pushtoons, 14%(54) Sindhi, 13%(50) Punjabi, 2.6%(10) Balochi, and remaining 18.7%(72) were of other ethnicities. High risk (≥7.5%) individuals were 20.7% (80/386) as per PCE and 11.1% (43/386) as per Astro-CHARM. As per Astro-CHARM, Sindhis’ had the least risk of ten-years ASCVD event among all the ethnicities, while, Urdu-speakings’ had the highest risk with mean rank of 145.18 vs. 216.50, p-value=0.001.

**Conclusion:**

A significant 10-years risk of first ASCVD event was observed in our population. ASCVD risk is alarmingly high in some ethnicities, such as Urdu-speaking, owing to the increased prevalence of traditional modifiable risk factors, such as diabetes and smoking.

## INTRODUCTION

About one-fourth of the world population, living in the South Asian region, are at an increased risk of atherosclerotic cardiovascular diseases (ASCVD).^1^,^2^ Worlds’ efforts to curtail the burden of ASCVD is paying off with the decline in the global burden of diseases, however, for the South Asian region it is on its rise.^3^ The increased risk of coronary artery (CAD) diseases among the South Asian population is ingrained in pathophysiological as well as lifestyle-related factors.^4^ A meta-analysis highlighted that the cross-sectional area of the coronary artery is smaller in South Asian population which increases the risk of CAD progression and development.^5^ Although, the biological mechanism of development of ASCVD is complex but increased prevalence of established risk factors in this population can be partly attributed to the increased risk of ASCVD.^1^

Pakistani population comprises of over 200 million inhabitants of the South Asian region. According to a recent study, more than 30% of the Pakistani individuals were at high risk (≥7.5%) of 10 years ASCVD events, smoking, diabetes (type II), hypertension, and high cholesterol were the most prevalent modifiable risk factors.^6^

Various risk assessment tools and clinical calculators have been developed for the assessment of ASCVD risk such as Framingham risk score, Reynolds risk score, systematic coronary risk evaluation, World Health Organization risk charts, Lancet chronic diseases risk charts, European system for cardiac operative risk evaluation (EuroSCORE), etc., but their clinical utility is limited due to poor concordance and predictability.^7^ According to the recent ACC/AHA primary prevention guidelines,^8^ individuals with an estimated risk of ≥ 7.5%, calculated based on the Pooled Cohort Equation (PCE), with coronary artery calcium (CAC) measured ≥ 100 Agatston units statin therapy should be initiated. The Astronaut Cardiovascular Health and Risk Modification (Astro-CHARM) risk calculator is an improvement over PCE with the addition of CAC and C-reactive protein (CRP).^9^

Hence, risk assessment is a vital step in implementing and developing preventive strategies. Available data elaborating risk of ASCVD for the Pakistani population and its ethnic breakup are based on traditional risk factors based calculators and data regarding CAC for our population is not available. The purpose of this study was to estimate the risk of atherosclerotic cardiovascular diseases (ASCVD) in various ethnicities of Pakistan using Astro-CHARM risk calculator and the Pooled Cohort Risk Assessment Equations.

## METHODS

This study was conducted at the National Institute of Cardiovascular Diseases (NICVD), Karachi, Pakistan during January to June 2019. Approval of the Ethical Review Committee (ERC-29/2018) was obtained and written informed consent was obtained from all the participants regarding the publication of their data while maintaining confidentiality. Both males and females of 40 to 65 years of age without pre-existing CAD were recruited using snowball sampling technique. Geographic coverage and ethnic distribution of the recruited individuals were ensured, detailed research methodology is presented elsewhere.^10^

The demographic data (gender, mother tongue, age (years), height (cm), and weight (kg)), lipid profile (total, HDL, and LDL cholesterol), systolic blood pressure measurement, and C-reactive protein (CRP) were obtained. History of the patients regarding traditional risk factors were obtained as per user guide of Astro-CHARM Calculator. Coronary computed tomography angiography (CCTA) was performed in all individuals to obtain coronary artery calcium (CAC) score (Agatston units).

Pakistani populations comprises of diverse racial and ethnical clusters dwelling in urban and rural areas. Ethnic distribution in rural areas are mostly outlined by administrative as well as geographic boundaries, nevertheless, the distribution in urban areas, especially in major cities, is not very well decisive.^11^ As, mother tongue (native language) is considered to be a strong surrogate of ethnicity, therefore, ethnicity of the participants was categorized as Sindhi, Punjabi, Pushtoon, Balochi, Urdu speaking (Muhajirs), and others (Sirayki, Bangali, Kashmiri, Gilgiti, etc.) based on the mother tongue.^12^ Ten-year risk of atherosclerotic cardiovascular diseases (ASCVD) event was estimated using Astro-CHARM Calculator^9^ (available at www.astrocharm.org) and Pooled Cohort Equation (PCE) Risk Assessment Equations^13^ (available at https://clincalc.com/cardiology/ascvd/pooledcohort.aspx). Variables included in the calculation of PCE and Astro-CHARM risk calculators are presented in Table-I.

**Table-I:** Variables included in the calculation of risk calculators.

Variables	Calculator	

	Astro-CHARM	Pooled Cohort Equation (PCE)
Gender	Included	Included
Age	Included	Included
Race	Included	Included
Total cholesterol	Included	Included
HDL cholesterol	Included	Included
Systolic blood pressure	Included	Included
Hypertension	Included	Included
Diabetes	Included	Included
Smoking	Included	Included
Family history of heart attack	Included	Not included
Coronary Artery Calcium	Included	Not included
C-reactive protein (CRP)	Included	Not included

The race variable was entered as “others” for Astro-CHARM Calculator and “white or other” for PCE. Participants were stratified into four groups based CAC score, no risk, low risk, medium risk, and high risk group with CAC score of 0 Agatston Units, 1 to 99 Agatston Units, 100 to 399 Agatston Units, and ≥ 400 Agatston Units respectively. Individuals with ≥ 7.5% of 10-years ASCVD risk based on Astro-CHARM and PCE were stratified as high risk.

All the data were collected on a predefined structural proforma entered on a data entry screen, developed using CsPro 7.0 by the U.S. Census Bureau and ICF International. Serpro S.A. Data were analyzed using International Business Machines (IBM) Statistical Package for the Social Sciences (SPSS), Version 21.0. Mean (± standard deviation), median [interquartile range (IQR)], minimum, and maximum of the ten-year atherosclerotic cardiovascular diseases (ASCVD) event risk were calculated for all the ethnicities. The Kolmogorov–Smirnov test (KS test) was applied for the calculated score of Astro-CHARM and PCE and hypothesis of normality of the distribution of calculated score was rejected. The Kruskal-Wallis test followed by pairwise comparisons were made to assess the calculated score by various ethnic groups. The p-value ≤ 0.05 was the set criteria for the statistical significance.

## RESULTS

In this study 386 individuals with mean age of 49 (±7.1) years without pre-existing CAD were included, out of which 45.3% (175) were females, 62.4% (241) were 40 to 50 years of age. The traditional risk factors of the cardiovascular diseases were as following; 45.6% (176) individuals were on antihypertensive treatment, 15.8% (61) were taking hyperglycemic treatments, 14.2% (55) were active smokers, and 30.6% (118) of these individuals had history of heart attack in their first degree relatives. The ethnic breakup was as follows; 33.4% (129) sample consisted of Urdu speaking individuals, 18.4% (71) were Pushtoon, 14% (54) were Sindhi, 13% (50) were Punjabi, 2.6% (10) were Balochi, and remaining 18.7% (72) of the individuals were of other ethnicities comprises of Sirayki, Bangali, Kashmiri, Gilgiti, etc. Demographic characteristics and distribution of traditional risk factors among the study participants by various ethnicities are presented in Table-II.

**Table-II:** Demographic characteristics and distribution of traditional risk factors among the study participants by various ethnicities.

Characteristics	Sindhi	Punjabi	Balochi	Pushto	Urdu	Others	p-value

	N=54	N=50	N=10	N=71	N=129	N=72	
Age (years)	47 (±7.3)	49.5 (±7.9)	46.9 (±8.7)	48.3 (±6.6)	50.3 (±6.8)	48.8 (±6.9)	0.019
40 to 50 years	42 (77.8%)	31 (62%)	7 (70%)	46 (64.8%)	67 (51.9%)	48 (66.7%)	0.029
51 to 65 years	12 (22.2%)	19 (38%)	3 (30%)	25 (35.2%)	62 (48.1%)	24 (33.3%)
***Gender***							
Male	33 (61.1%)	28 (56%)	3 (30%)	31 (43.7%)	73 (56.6%)	43 (59.7%)	0.168
Female	21 (38.9%)	22 (44%)	7 (70%)	40 (56.3%)	56 (43.4%)	29 (40.3%)
***Medical History***							
Hypertension	26 (48.1%)	23 (46%)	5 (50%)	39 (54.9%)	47 (36.4%)	36 (50%)	0.177
Diabetes	2 (3.7%)	6 (12%)	2 (20%)	14 (19.7%)	26 (20.2%)	11 (15.3%)	0.098
Currently Smoke	6 (11.1%)	7 (14%)	1 (10%)	6 (8.5%)	20 (15.5%)	15 (20.8%)	0.385
FHxHA	10 (18.5%)	19 (38%)	4 (40%)	21 (29.6%)	49 (38%)	15 (20.8%)	0.035

FHxHA = family history of heart attack.

**Fig.1:**
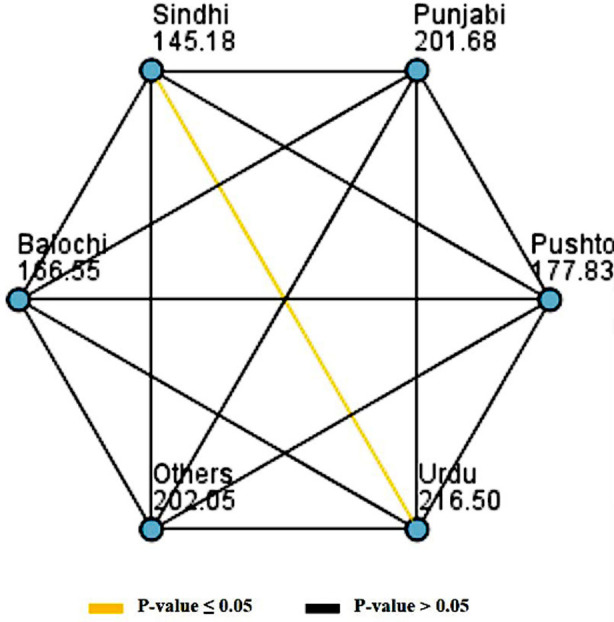
The pairwise comparisons of ten-year risk of atherosclerotic cardiovascular diseases (ASCVD) by ethnicities.

The summary of ten-year risk of atherosclerotic cardiovascular diseases (ASCVD) event estimated using Astro-CHARM and Pooled Cohort Equation (PCE) for the ethnicities are presented in Table-III. The Kruskal-Wallis test revealed that the distribution of risk calculated using the Pooled Cohort Equation (PCE) is same across the ethnicities with p-value of 0.061 (Chi-square = 10.538, degrees of freedom = 5). While, the distribution of risk calculated using Astro-CHARM calculator is not same across the ethnicities with p-value of 0.003 (Chi-square = 18.301, degrees of freedom = 5). The pairwise comparison of the calculated risk among the various ethnicities showed that the “Sindhi” had the least risk of ten years ASCVD event among all the ethnicities, while, “Urdu Speaking” had the highest risk of ten years ASCVD event with mean rank of 145.18 vs. 216.50, p-value = 0.001. The pairwise comparisons of ten-year risk of atherosclerotic cardiovascular diseases (ASCVD) by ethnicities are presented in Fig.1.

**Table-III:** The summary of ten-year risk of atherosclerotic cardiovascular diseases (ASCVD) event estimated using Astro-CHARM and Pooled Cohort Equation (PCE) for the ethnicities.

Ethnicities	Base	10-years ASCVD Event Risk (%)			

	N	Mean (± SD)	Median [IQR]	Range	Mean Rank
***Astro-CHARM***					
Total	386	3.57 (± 4.62)	1.9 [4 - 1.1]	40.3 - 0.2	-
Sindhi	54	1.88 (± 1.59)	1.4 [2.2 - 0.9]	9 - 0.3	145.18
Punjabi	50	4.08 (± 5.43)	1.9 [5.4 - 1.3]	31.4 - 0.5	201.68
Balochi	10	2.13 (± 1.56)	2 [2.6 - 1]	5.9 - 0.6	166.55
Pushto	71	2.74 (± 2.82)	1.8 [3.5 - 1]	17.7 - 0.2	177.83
Urdu	129	4.62 (± 5.78)	2.4 [5.7 - 1.2]	40.3 - 0.5	216.50
Others	72	3.6 (± 4.42)	2.45 [4.05 - 1.15]	25 - 0.4	202.05
***Pooled Cohort Equation (PCE)***					
Total	386	4.96 (± 5.79)	2.8 [6.2 - 1.3]	43.8 - 0.2	-
Sindhi	54	2.99 (± 2.77)	2.1 [4.2 - 1.1]	12.1 - 0.2	156.89
Punjabi	50	5.16 (± 5.84)	2.95 [6.8 - 1.4]	25.1 - 0.5	197.47
Balochi	10	4.03 (± 5.06)	2.45 [5.4 - 1]	17.5 - 0.5	168.65
Pushto	71	4.15 (± 4.52)	2.7 [5.7 - 1.1]	26.1 - 0.4	182.21
Urdu	129	5.83 (± 6.36)	3.7 [7 - 1.7]	33.4 - 0.3	210.43
Others	72	5.67 (± 7.1)	3 [7 - 1.6]	43.8 - 0.5	202.45

According to PCE, 20.7% (80/386) of individuals were at high risk (≥ 7.5%) of 10-years ASCVD event. Burden of high risk of 10-years ASCVD event by the ethnicities are as following; rates of high risk were 11.1% (6/54) for Sindhi, 24% (12/50) for Punjabis, 10% (1/10) for Balochi, 16.9% (12/71) for Pushtoon, 24.8% (32/129) for Urdu speaking individuals and 23.6% (17/72) for other ethnicities. Similarly, according to Astro-CHARM, 11.1% (43/386) were high risk (≥ 7.5%) individuals, rates of high risk were 1.9% (1/54) for Sindhi, 14% (7/50) for Punjabi, 0% (0/10) for Balochi, 7% (5/71) for Pushtoon, 18.6% (24/129) for Urdu speaking individuals and 8.3% (6/72) for other ethnicities.

No coronary artery calcium was observed in 73.1% (282) of the individuals, 20.5% (79) were in the low-risk groups with CAC score of 1 to 99 Agatston Units, 4.9% (19) were in moderate-risk groups with CAC score between 100 and 399 Agatston Units, and remaining 1.6% (6) individual were in high-risk group with CAC score ≥ 400 Agatston Units. Distribution of the coronary artery calcium (CAC) score for the various ethnicities are presented in Table-IV.

**Table-IV:** Distribution of the coronary artery calcium (CAC) score for the various ethnicities.

CAC (Agatston Units)	Sindhi	Punjabi	Balochi	Pushto	Urdu	Others

	N=54	N=50	N=10	N=71	N=129	N=72
No risk – (0)	46 (85.2%)	34 (68%)	8 (80%)	58 (81.7%)	81 (62.8%)	55 (76.4%)
Low risk – (1 to 99)	6 (11.1%)	10 (20%)	2 (20%)	11 (15.5%)	36 (27.9%)	14 (19.4%)
Moderate risk – (100 to 399)	2 (3.7%)	4 (8%)	0 (0%)	1 (1.4%)	9 (7%)	3 (4.2%)
High risk – (≥ 400)	0 (0%)	2 (4%)	0 (0%)	1 (1.4%)	3 (2.3%)	0 (0%)

CAC = coronary artery calcium score.

## DISCUSSION

This is the first study from Pakistan, or even in the South Asian region, reporting 10 years ASCVD risk estimates using Astro-CHARM calculator. Pakistani inhabitants are multicultural with each sub-culture and ethnicity having distinctive lifestyle, socio-economic, and cultural values.^14^ Therefore, it was deemed important to assess the risk of atherosclerotic cardiovascular diseases among the various ethnicities dwelling in Pakistan. Low to middle socio-economic class dominates the Pakistani population landscape with a diverse cultural and ethnic breakup. Studies in the past reported great variations in the incidence and risk of ASCVD among the various ethnic breakups of Pakistan.^10^,^12^,^15^

We observed that our Urdu speaking community (commonly known as Muhajirs) is at increased risk of development of ASCVD event in the following 10 years period and presence of coronary artery calcium was also higher in this community. Diabetes, smoking, and family history of heart attack was relatively more common in Urdu speaking communities as compared to the other ethnic groups. In this study individuals of Sindhi ethnic origin were found to have least estimated risk of 10 years ASCVD event with least diabetics of all the other ethnicities. Punjabi are the second most risky ethnicity with relatively high prevalence of traditional risk factors.

These findings are aligned with the findings of a past study by Ashraf T et al.^12^, in their study of non-atherosclerotic Pakistani individuals, 44.87% of Urdu speaking had ≥ 7.5% risk of atherosclerotic event in 10 years followed by Punjabi with 19.23% and high-risk individuals were in the smallest ratio for Sindhi ethnicity. This study along with other studies in this population,^6^,^8^,^10^,^12^ estimates a significant risk of ASCVD in Pakistani population, and for subgroups, such as Urdu speaking and Punjabi, it is alarmingly high.

The coronary artery calcium (CAC) is one of the robust independent predictor and a novel surrogate marker of subclinical ASCVD.^16^-^18^ Progression in the repeated assessment of CAC on computed tomography (CT) scan was reported to be associated with the progression and incidence of CAD and mortality.^19^-^21^ For the adult population risk of fatal and nonfatal cardiac events observed to be associated with the presence of CAC.^22^,^23^ Considering the strong predictability, traditional risk factors based risk assessment calculators^13^ were upgraded by integrating CAC score with the conventional risk factors to form a robust risk stratification modality for the ASCVD,^9^ and the recent 2019 ACC/AHA guidelines for the primary prevention of CAD recommended ASCVD risk calculation and CAC measurement to guide the statin therapy.^8^

A systematic risk assessment and initiations of preventive measures including lifestyle modification and lipid-lowering medications are the need of the hour for our population. However, neither Astro-CHARM calculator nor PCE are validated for this population, therefore, future research efforts are also needed to optimize the risk assessment and stratification methods.

A popular saying of “diversity is strength” doesn’t seem to favor the Pakistani population when it comes to ASCVD risk assessment and prevention. With a bigger aim of reduction of ASCVD burden at national as well as regional level, it is important to study the sub-clinical and clinical factors in the various ethnicities at its native grounds and propagate the targeted preventive strategies. Secondly, awareness campaigns and assurance of adaptation of evidence-based medicine and guidelines need to be ensured not only in cardiology clinics but also at the general practitioners’ office in urban as well as far-flung rural areas of Pakistan.

### Limitations of the study

The Snowball sampling technique adopted for participant recruitment is the limitation of the study, secondly, ethnic distribution of study sample was not as per population distribution of Pakistan, hence, the generalizability of results is limited. Categorization of ethnicities based on mother tongue may does not reflect true ethnicity especially for “Urdu speaking”.

## CONCLUSION

In the present study, we observed a significant estimated 10 years risk of first atherosclerotic cardiovascular disease (ASCVD) in Pakistani individuals of diverse ethnic backgrounds without preexisting cardiovascular diseases. The risk of ASCVD is alarmingly high for some of the ethnic groups, such as Urdu speaking, owing to the increased prevalence of traditional modifiable risk factors, such as diabetes and smoking.

### Authors’ Contribution:

**TA:** Conceived the idea, **TA, MK & AN** Designed the study, **TA, AN & SS:** Collected the data, **TA & MK:** Drafted the manuscript, **AN & SS:** Critically reviewed the manuscript.

**TA:** Agrees to be accountable for all aspects of work ensuring integrity and accuracy.
